# Review and Long-Term Outcomes of Cruciate Ligament Reconstruction versus Conservative Treatment in Siblings with Congenital Anterior Cruciate Ligament Aplasia

**DOI:** 10.1155/2017/1636578

**Published:** 2017-05-14

**Authors:** Diego Davanzo, Paolo Fornaciari, Geoffroy Barbier, Mauro Maniglio, Daniel Petek

**Affiliations:** ^1^Clinic of Orthopedic Surgery, Kantonsspital Fribourg, Chemin des Pensionnats 2-6, 1708 Fribourg, Switzerland; ^2^Department of Orthopaedics, Balgrist University Hospital, Forchstrasse 340, 8008 Zürich, Switzerland

## Abstract

There is no consensus on the best treatment for anterior cruciate ligament hypoplasia or aplasia. To our knowledge, no comparative study between operative and conservative treatment of this condition has ever been performed. Conservative treatment is a viable alternative to surgery for ACL aplasia. Two siblings were examined at our outpatient clinic. The male patient underwent bilateral ACL reconstruction, while his sister was treated conservatively. Our results show a worse long-term outcome for the operative patient. At her last follow-up, the female patient treated conservatively showed subjective improvement in stability and gait. A review of the literature shows inconsistent outcomes after reconstruction in contrast to reports with cruciate ligament agenesis that did not undergo reconstruction with acceptable to good outcomes. Cruciate reconstruction should be reserved for cases of impaired articular instability, objectively manifest in the frequency of giving-way episodes. Treatment depends on the patient's condition and expectations. Surgery should therefore only be suggested after proper patient counseling.

## 1. Introduction

Hypoplasia or aplasia of the cruciate ligaments is a rare congenital condition, with a prevalence of 0.017 per 1000 live births [1]. The first well-documented report on the congenital absence of the anterior cruciate ligament was made by Katz et al. [2] in 1967. The familial character of the pathology without an associated lesion was first studied by Frikha et al. [3], who suggested that the pathology is hereditary and transmitted in an autosomal dominant pattern.

The pathophysiology of cruciate ligament aplasia and its impact on daily life are not well understood, and clinical management remains controversial. To our knowledge, no comparative study between operative and conservative treatment of cruciate ligament aplasia in siblings has been performed. The purpose of this case report is to review our experience of the treatment of two siblings with a bilateral symptomatic congenital absence of the anterior cruciate ligament (ACL). We will also present a review of the literature.

## 2. Case 1

**Figure 1:**
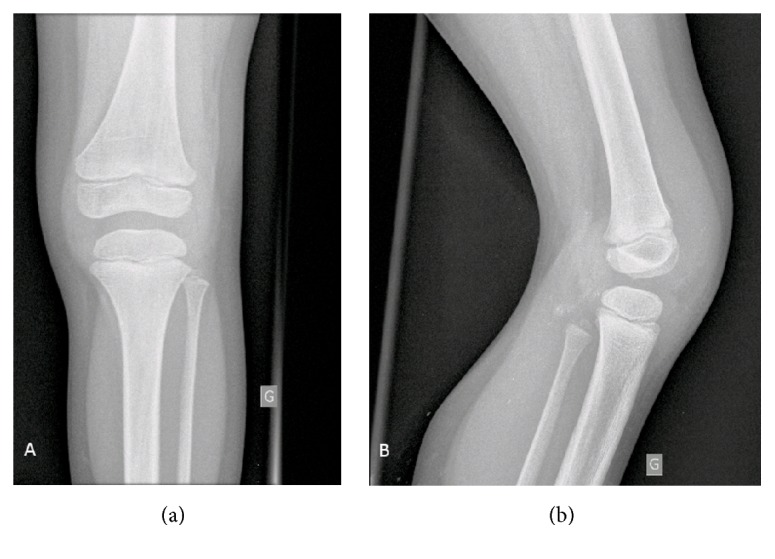
Plain, anteroposterior (a) and lateral (b) radiographs of a 6-year-old male patient's left knee showing hypoplasia of the tibial intercondylar eminence and of the femoral intercondylar notch.

**Figure 2:**
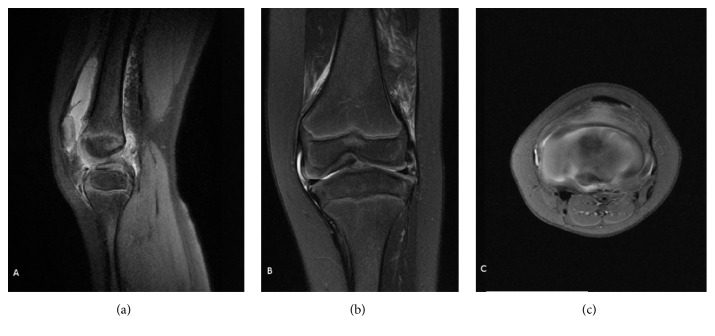
Preoperative MRI of the left knee of the patient from Figure 1 (sagittal (a), coronal (b), and transverse (c) views). Complete agenesis of the ACL and PCL hypoplasia is noted. The femoral intercondylar notch is completely covered by cartilage. The tibial spine is hypoplastic.

**Figure 3:**
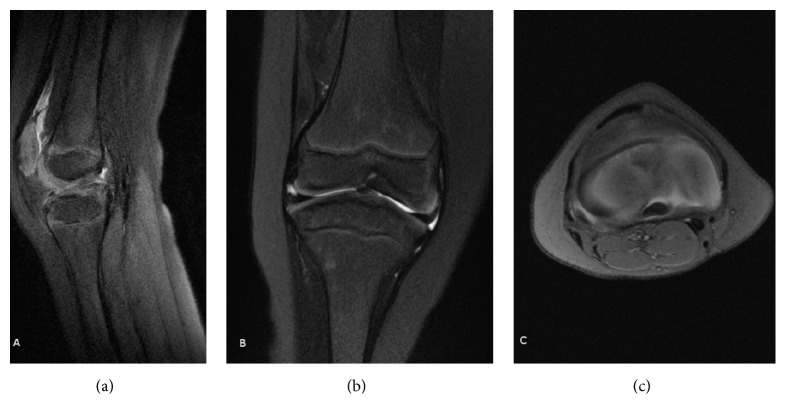
Preoperative MRI (sagittal (a), coronal (b), and transverse (c) views) of the right knee of the patient from Figures 1 and 2 showing the same findings as those in the left knee.

A 6-year-old male patient was evaluated at our center for a history of left knee pain after a sprain. Physical examination revealed a limping gait intra-articular swelling and a bilateral anterior instability of both knee joints. The modified Lysholm score was 55 on the left knee and 66 on the right knee. Standard radiographs showed hypoplasia of the tibial intercondylar eminence and the femoral intercondylar notch (Figures 1(a) and 1(b)). Bilateral magnetic resonance images (MRI) showed complete agenesis of the ACL and hypoplasia of the posterior cruciate ligament (PCL). The femoral intercondylar notch was shallow and completely covered by cartilage. Tibial spine hypoplasia was also present (Figures 2(a)–2(c) and 3(a)–3(c)). Staged surgical treatment of both knees with cruciate ligament reconstruction was proposed.

The patient underwent arthroscopic evaluation that confirmed an aplasia of the ACL (Manner II [1]), a hypoplasia of the PCL, and a dysplastic intercondylar notch. A reconstruction of the ACL with autologous patellar tendon grafting with epiphyseal femoral fixation and transepiphyseal tibia fixation associated with a modified MacIntosh procedure (Figures 8(a)–8(c)) was performed by cutting a strip of the tractus iliotibialis which was sutured to Gerdy's tubercle after encircling the lateral collateral ligament. During the arthroscopy, the presence of an atypical meniscus with a prolongation of the posterior horn to the insertion of the ACL was remarked. The lateral meniscus was ring-shaped with invasion of the central compartment. The lateral femoral condyle showed grade II and III Outerbridge lesions.

The autologous patellar tendon graft was harvested without a bony segment. The range of motion was tested up to a flexion and extension of 90°/0°/0°. At this point the stalked graft was passed from Gerdy's tubercle behind the lateral collateral ligament and the popliteal tendon and sutured to the lateral femoral condyle. A splint with a flexion of 20° was adapted and the leg was passively mobilized for four weeks. Full weight-bearing was started at eight postoperative weeks. Physical therapy was prolonged for 5 months because of a moderate limitation in knee extension which caused a limping gait. Nine months after the operation of the left knee the patient was free of symptoms. The operation on the right knee consisted of a diagnostic arthroscopy with reconstruction of the ACL with autologous semitendinosus and gracilis tendon grafting along with epiphyseal femoral and transepiphyseal tibial fixation. A MacIntosh lateral ligamentoplasty was performed at the same time.

**Figure 4:**
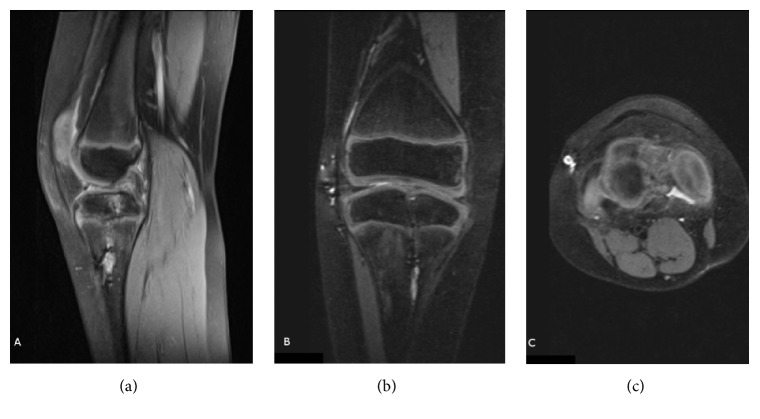
Postoperative MRI (sagittal (a), coronal (b), and transverse (c) views) of the left knee of the patient from Figures 1–3. Graft integrity was inconclusive.

**Figure 5:**
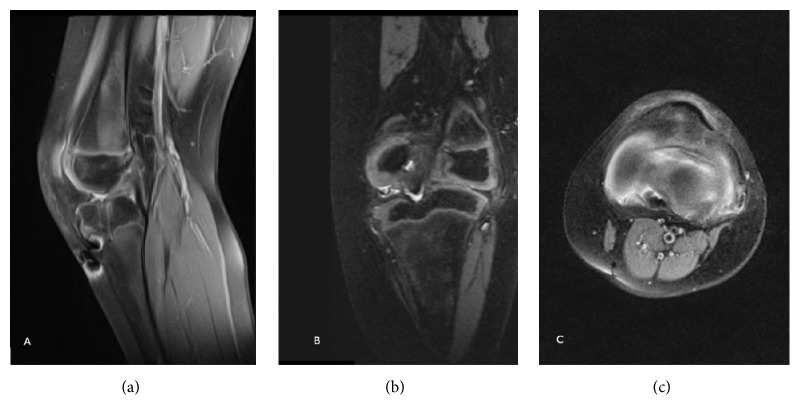
Postoperative MRI (sagittal (a), coronal (b), and transverse (c) views) of the right knee of the patient from Figures 1–4. Graft integrity was inconclusive.

Forty months after the first operation, the patient was not impaired in his daily activities but displayed bilateral laxity on physical examination. The patient suffered from frequent episodes of his left knee giving way, with associated swelling and tenderness. Bilateral MRIs (Figures 4(a)–4(c) and 5(a)–5(c)) were inconclusive with respect to ACL graft integrity on the left side. The patient's symptoms decreased with physical therapy. Physical examination findings 5 years after surgery are described in Table 1. His modified Lysholm score was 53 for the right knee and 49 for the left knee. The patient had limited daily ambulation but continued recreational football activities with clear limitation and aches.

## 3. Case 2

**Figure 6:**
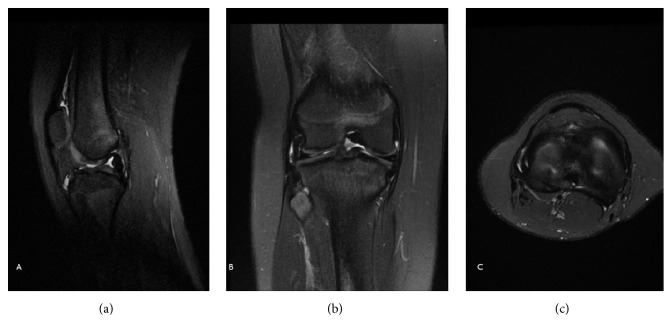
MRI (sagittal (a), coronal (b), and transverse (c) views) of the right knee of a 15-year-old female patient shows complete agenesis of the ACL and a normal PCL.

**Figure 7:**
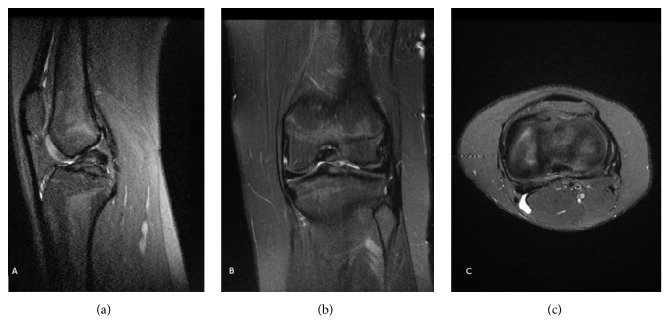
MRI (sagittal (a), coronal (b), and transverse (c) views) of the left knee of the patient from Figure 6 showing the same findings as her right knee.

**Figure 8:**
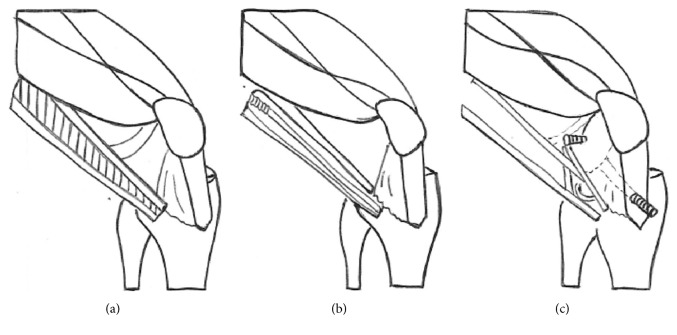
Modified MacIntosh procedure for ACL reconstruction.

The 15-year-old girl sister of the first patient was also examined at our outpatient clinic for occasional knee pain, swelling, and functional instability of both knees under pivot stress. Her symptoms started 1 year earlier and were present inconsistently during school physical exercise. Her modified Lysholm score was estimated to be 52 on both knees. Both knees had a positive anterior drawer test. Bilateral MRIs showed complete agenesis of the ACL but a normal PCL (Figures 6(a)–6(c) and 7(a)–7(c)). Training and functional therapy as well as proprioceptive exercises were started.

At the 2-year follow-up, the patient showed improved subjective stability and gait. The findings are summarized in Table 1. Her modified Lysholm score was 91 bilaterally. The patient was not limited in daily walking, found employment after graduation, and did not engage in any kind of athletic activity.

## 4. Discussion

The first report of congenital cruciate ligament agenesis was published by Giorgi [4] in 1957. Familial presentation of this pathology was rarely described in the English literature [3, 5, 6], even if heritability was suspected. According to Frikha et al. [3], congenital cruciate ligament agenesis is autosomal dominantly transmitted with variable prevalence. According to Steckel et al. [6], the presence of a lateral discoid meniscus is associated with ACL aplasia, as these structures develop during the same embryologic stage [6, 7]. Bilateral ACL agenesis has been associated with other abnormalities, including proximal focal femoral deficiency [1], tibial or fibular dysplasia [8], subluxation or dislocation of the patella [8], ligament hyperlaxity [8], congenital talipes equinovarus, fibular hemimelia [1], agenesis or hypoplasia of the patella [9], hip dysplasia or dislocation [9], and scoliosis [10].

Manner et al. [1] analyzed 34 knees in 31 patients and identified three types of cruciate ligament aplasia. Type I aplasia has a hypoplastic or aplastic ACL and a normal PCL. The notch width index is reduced, and the medial tibial spine is normal. Type II aplasia involves the ACL and PCL, with a decreased notch width index, aplastic lateral tibial spine, and hypoplastic medial tibial spine. Type III aplasia is marked by ACL and PCL aplasia, an absent intercondylar notch with hyaline cartilage in its place and aplastic tibial spines.

The clinical symptoms of cruciate ligament aplasia can range from completely asymptomatic [11] to severely incapacitating and may be associated with congenital knee dislocation [9]. Functional instability is the main complaint of patients with cruciate ligament aplasia. This instability may lead to a slowly progressive osteoarthritis of later onset compared with traumatic ACL injuries [3]. As suggested by Steckel et al. [6, 8], the absence of the cruciate ligaments is probably balanced by muscle compensation. However, decompensation of this balance after trauma can lead to symptom development.

The first report of ACL agenesis treated with autologous reconstruction was presented by Katz et al. in 1967 [2]. Two boys and one girl with a total of five ACL-deficient knees were surgically treated with a modified Jones technique. One of the five knees required a high tibial osteotomy because of redislocation. The authors reported good operative outcomes in all cases.

Kaelin et al. [12] documented arthroscopically six knees with ligamentous aplasia. Two knees underwent a concomitant meniscectomy, and one also required a lateral Lemaire stabilization. The case treated only with meniscectomy had a good outcome, while the patient who required Lemaire stabilization—despite improved stability clinically—did not improve subjectively.

Dejour et al. [7] described a 34-year-old female with bilateral ligamentous aplasia treated bilaterally with ACL reconstruction with patellar autograft, anteroexternal Lemaire ligamentoplasty, internal ligamentoplasty with lateral ligament tensioning, posterointernal ligamentoplasty, and tibial osteotomy to correct an extension defect. At the 3-year follow-up after the first operation and the 2-year follow-up after the second operation, the patient was generally satisfied with her outcome and denied subjective instability. However, she had persistent pain in the second operated knee.

The study by Gabos et al. [13] describes four patients who underwent tendon allograft ACL reconstructions. An isolated arthroscopically assisted ACL reconstruction was performed in two cases. The other two patients underwent open ACL reconstruction through old scars. One of these latter patients underwent a patella tendon allograft ACL reconstruction, followed by open posterolateral corner reconstruction using double-limbed bone. An Achilles tendon allograft was used to recreate the popliteus and popliteofibular ligament, while an inferior slip of autologous biceps tendon was used to recreate the lateral collateral ligament. The other patient from the open ACL group required osteochondral defect reconstruction. After an average follow-up of 31 months between the four patients, only one case of a 10-degree extension deficit was reported in the arthroscopic reconstruction group. Laxity was clinically and subjectively improved in all patients, with a mean Lysholm score of 81.

Steckel et al. [6] reported a case of ACL and PCL absence treated with ACL reconstruction with autologous patellar tendon graft. The patient still complained of instability postoperatively, and posterior subluxation of the tibia was evident. Symptoms resolved only after ACL graft resection and intensive physical therapy.

Knorr et al. [14] reported a case of a 3-year-old symptomatic boy with ACL agenesis and a hypoplastic medial meniscus. He underwent a Clochville ACL reconstruction using an autologous patellar graft at the age of 5. MRI imaging at the 4-year follow-up showed complete graft integration. At the 5-year follow-up, the patient was asymptomatic (Lysholm score of 99), with clinically improved stability.

Knorr et al. [14] described the case of a symptomatic 21-year-old patient with ACL agenesis and a hypoplastic PCL. She underwent a notchplasty and a transtibial single-bundle ACL reconstruction with anterior tibialis tendon allograft. At the 6-month follow-up, the patient showed improved clinical stability without range of motion restriction.

Bedoya et al. [10] treated a 13-year-old girl with knee pain and instability. A thickened PCL was associated with ACL agenesis. ACL reconstruction using a posterior tibial tendon allograft was performed on the left knee. The patient was stable at follow-up and underwent surgery on her right knee 7 months later with the same procedure. At her 2-year follow-up, she denied instability.

A study by Sonn and Caltoum [5] reported on 15-year-old male monozygotic twins with symptomatic ACL agenesis. Both underwent ligament reconstruction with a patellar graft. At 32 months postoperatively, both patients had good clinical knee stability and resumed their previous athletic activities.

One of the largest series on congenital cruciate ligament agenesis was reported by Thomas et al. [15]. In this series, half of the patients were asymptomatic, while others admitted to their knee giving way more than once a week. All patients underwent arthroscopy, but no ACL reconstruction was performed.

Past reports in the literature of ACL aplasia are listed and described in Table 2.

Many other reports show acceptable to good outcomes of patients with cruciate ligament agenesis who do not undergo reconstruction [3, 16–20], in contrast to the inconsistent outcomes reported after reconstruction.

In the first months after surgery, the stability of the boy's knees objectively improved. However, after a few years his instability recurred, with symptoms of giving way and poor functional outcome. His sister had a better result with muscle reinforcement through physical therapy, which yielded long-lasting benefits. The number of cases in our study was limited by the rarity of the disease.

Here, we show an attempt to stabilize both knees in a 6-year-old boy with ACL aplasia. The risk of surgical failure was high from the onset, due to the fact that the graft could not follow the patient's skeletal growth. Moreover valgus deformities of the knee, leg length discrepancies, and hip or ankle dysplasia are frequently associated with cruciate ligament aplasia. A surgical approach is also reasonable if epiphyseal closure is not yet obtained but should be attentively considered. A study of Holwein et al. [21] reported that transphyseal ACL reconstruction with metaphyseal fixation in children with open growth plates can be done with low risk of growth changes, and return to competitive sports is possible although low rotational laxity still exists. However the study of Collins et al. [22] reported that growth abnormalities after ACL reconstruction in the skeletally immature patient are underreported, and our current understanding of the etiology of these abnormalities is limited.

Based on our literature review, most symptomatic cases of ligamentous aplasia start with a low-grade trauma or sprain and end in muscular imbalance. A long-lasting symptom history is not necessarily the rule in this pathology. Sufficient time should be allocated to assess the benefits of physical rebalancing before surgical reconstruction is considered. We agree with the statements of de Ponti et al. [8] that the management of ligamentous aplasia should take into account factors such as age, type and level of activity, and quality and amount of symptoms. Cruciate reconstruction should be reserved for cases of impaired articular stability, which is objectively manifest in frequent giving-way episodes. The indication for surgery is also based on the patient's condition and expectations and should only be considered after proper counseling.

Our analysis of the literature illustrates the current disagreement on the appropriate treatment for congenital ligamentous aplasia. Further studies using internationally recognized assessments are needed to create a widely accepted treatment algorithm.

## Figures and Tables

**Table 1:** Bilateral clinical outcomes of both patients at their last follow-up.

Last follow-up	Case 1		Case 2	
	Right	Left	Right	Left
Modified Lysholm score	53	49	91	91
Femoral axis	5° valgus	5° valgus	0°	0°
ROM F/E	150/0/5°	150/0/5°	150/0/5°	150/0/5°
Anterior drawer test (rolimeter mm)	12	18	10	8
Lachmann test	III	III	I	I
Pivot shift	pos	pos	neg	neg
Posterior drawer test	pos	neg	neg	neg
Varus instability 0° (mm)	10	5	<5	<5
Valgus instability 0° (mm)	5	5	<5	<5
Varus instability 30° (mm)	15	5	<5	<5
Valgus instability 30° (mm)	15	15	10	5

**Table 2:** Summary of published studies regarding the treatment of ACL aplasia.

Author	Publication year	Number of knees	Description
Katz et al.	1967	5	ACL agenesis treated with autologous reconstruction with good operative outcomes in all cases.

Thomas et al.	1985	12	Congenital cruciate ligament agenesis: half of the patients were asymptomatic, while the other half admitted to their knee giving way more than once a week. All patients underwent arthroscopy, but no ACL reconstruction was performed.

Kaelin et al.	1986	6	Documentation of arthroscopy in six knees with ligamentous aplasia.

Dejour et al.	1990	1	Bilateral ligamentous aplasia treated bilaterally with surgery. There was no subjective instability. However, there was persistent pain in the second operated knee.

de Ponti et al.	2001	1	Bilateral ACL agenesis is associated with tibial or fibular dysplasia, subluxation, or dislocation of the patella, ligament hyperlaxity.

Gabos et al.	2005	4	Four patients who underwent tendon allograft ACL reconstructions. After a follow-up of 31 months, only one case of a 10-degree extension occurred. Laxity was clinically and subjectively improved in all patients, with a mean Lysholm score of 81.

Frikha et al.	2005	8 knees in the same family	Congenital cruciate ligament agenesis is autosomal, dominant.

Steckel et al.	2005	1	Lateral discoid meniscus is associated with ACL aplasia. Case of ACL and PCL absence treated with surgery.

Manner et al.	2006	34	Bilateral ACL agenesis is associated with proximal focal femoral deficiency, congenital talipes equinovarus, fibular hemimelia.

Knorr et al.	2006	1	Case of ACL agenesis and a hypoplastic medial meniscus treated with Clochville ACL reconstruction. At the 5-year follow-up, the patient was asymptomatic, with clinically improved stability.

Lee et al.	2006	1	Case of ACL agenesis and a hypoplastic PCL treated with notchplasty and transtibial single-bundle ACL reconstruction. At the 6-month follow-up, improved clinical stability without range of motion restriction was observed.

Roth et al.	2010	3	Bilateral ACL agenesis is associated with agenesis or hypoplasia of the patella, hip dysplasia, or dislocation.

Bedoya et al.	2014	1	Case of ACL agenesis treated with surgery. Bilateral ACL agenesis is associated with scoliosis.

Sonn and Caltoum	2014	1	Male monozygotic twins with symptomatic ACL agenesis. Both underwent ligament reconstruction with a patellar graft. At 32 postoperative months, both patients had good clinical knee stability.

## Abbreviations

ACL: Anterior cruciate ligament
PCL: Posterior cruciate ligament.
